# Bilateral Sensory Abnormalities in Patients with Unilateral Neuropathic Pain; A Quantitative Sensory Testing (QST) Study

**DOI:** 10.1371/journal.pone.0037524

**Published:** 2012-05-22

**Authors:** Karl-Heinz Konopka, Marten Harbers, Andrea Houghton, Rudie Kortekaas, Andre van Vliet, Wia Timmerman, Johan A. den Boer, Michel M.R.F. Struys, Marten van Wijhe

**Affiliations:** 1 Pain Management Unit,Department of Anesthesiology, University Medical Center Groningen, University of Groningen, Groningen, The Netherlands; 2 Department of Psychiatry, University Medical Center Groningen, University of Groningen, Groningen, The Netherlands; 3 Merck and Company Incorporated, West Point, New York, United States of America; 4 Department of Neuroscience, Neuroimaging Center, University Medical Center Groningen, University of Groningen, Groningen, The Netherlands; 5 PRA International, Zuidlaren, The Netherlands; The James Cook University Hospital, United Kingdom

## Abstract

In patients who experience unilateral chronic pain, abnormal sensory perception at the non-painful side has been reported. Contralateral sensory changes in these patients have been given little attention, possibly because they are regarded as clinically irrelevant. Still, bilateral sensory changes in these patients could become clinically relevant if they challenge the correct identification of their sensory dysfunction in terms of hyperalgesia and allodynia. Therefore, we have used the standardized quantitative sensory testing (QST) protocol of the German Research Network on Neuropathic Pain (DFNS) to investigate somatosensory function at the painful side and the corresponding non-painful side in unilateral neuropathic pain patients using gender- and age-matched healthy volunteers as a reference cohort. Sensory abnormalities were observed across all QST parameters at the painful side, but also, to a lesser extent, at the contralateral, non-painful side. Similar relative distributions regarding sensory loss/gain for non-nociceptive and nociceptive stimuli were found for both sides. Once a sensory abnormality for a QST parameter at the affected side was observed, the prevalence of an abnormality for the same parameter at the non-affected side was as high as 57% (for Pressure Pain Threshold). Our results show that bilateral sensory dysfunction in patients with unilateral neuropathic pain is more rule than exception. Therefore, this phenomenon should be taken into account for appropriate diagnostic evaluation in clinical practice. This is particularly true for mechanical stimuli where the 95% Confidence Interval for the prevalence of sensory abnormalities at the non-painful side ranges between 33% and 50%.

## Introduction

In clinical practice, the assessment of chronic pain includes documentation of pain location, intensity, quality and onset/duration aimed to elucidate the underlying pathophysiological mechanism. Sensory testing is an important part of this assessment which is aimed at identifying phenomena such as hyperalgesia (increased response to painful stimuli) and allodynia (painful response to normally non-painful stimuli) for thermal and mechanical stimuli [1]. For this clinical evaluation, patients are generally used as their own control when comparing profiles of sensory dysfunction at the painful side with the contralateral non-painful area [1], [2]. The correct identification of the specifics of sensory dysfunction in each chronic pain patient is obviously of major importance for addressing the underlying mechanism such as peripheral or spinal hyperexcitability and has consequences for pharmacological treatment.

There are only a few studies reporting bilateral sensory abnormalities in chronic pain conditions. Huge and co-workers, 2008 investigated thermal sensory function at the affected and non-affected side of acute and chronic complex regional pain syndrome (CRPS) patients and found bilateral sensory changes for both patient groups [3]. Another study investigating bilateral warmth/cold detection and heat/cold pain thresholds over the hand/wrist in patients with unilateral carpal tunnel syndrome (CTS) revealed bilateral thermal hyperalgesia in patients with strictly unilateral CTS compared to controls [4]. In a similar patient population, Fernández-de-las-Peñas and colleagues (2009) reported bilateral pressure pain hyperalgesia in patients with unilateral CTS [5].

In spite of the studies referred to above, the occurrence of contralateral sensory changes in situations where the pain is experienced only unilaterally is still not generally acknowledged. Possibly this is because it is regarded clinically irrelevant. However, bilateral sensory changes could become clinically relevant in patients with unilateral pain if they challenge the correct qualification of sensory dysfunction. For example, if a mechanical stimulus which is known to be slightly painful presented at the non-affected and affected side is rated by the patient as equally painful at both sides, one could conclude normal sensory functioning. However, if both the non-affected and affected side of this patient are hyperalgesic for this particular stimulus, the conclusion of a mechanical hyperalgesia could be overseen.

As the neuropathic pain is characterized by both, positive and negative sensory phenomena, it is critical for those phenomena to be captured and, for their optimal utility, to be measured quantitatively. The German Research Network on Neuropathic Pain (DNFS) established a standardized Quantitative Sensory Testing (QST) protocol which allows a comprehensive somatosensory characterisation of chronic neuropathic pain patients, using reference values from healthy volunteers [6], [7]. This protocol uses 13 different mechanical and thermal stimuli (e.g. graded von Frey filaments, pin-prick devices, a pressure algometer, and quantitative thermo-testing). It takes about 30 minutes to test one location of the body in healthy volunteers and about 45 minutes in patients. This QST battery tests different sub-modalities of nerve fibres involved in the transduction of sensory information from the periphery to the spinal cord such as Aβ-fibre, Aδ-fibre and C-fibre [6], [7].

There is a long tradition of quantitative measurement of somatic sensory function, well documented in a number of publications [8], [9], [10], [11] and it has been shown to be adequate with respect to reliability and validity [12]. Several publications show that also QST is valid, reliable and sensitive to quantify sensory abnormalities [13], [14], [15], [16].

By using reference values from healthy volunteers, QST does not rely on reference values obtained from the patient’s own contralateral side. Thus, it offers a unique opportunity to study bilateral somatosensory function in patients with chronic unilateral pain in a detailed, standardized manner.

Based on previous reports we hypothesize that bilateral somatosensory abnormalities are frequently present in unilateral chronic pain patients and that bilateral sensory changes may exist for the same QST parameter. To test this we selected a large cohort of patients with unilateral neuropathic pain. We examined the painful side and its corresponding contralateral area using the standardized DNFS QST protocol comparing values with those obtained from age- and gender-matched healthy volunteers.

## Methods

### Ethics Statement

The study adhered to the declaration of Helsinki and was approved by the independent, medical ethical committee “Stichting Beoordeling Ethiek Bio-Medisch Onderzoek”, P.O. Box 1004, 9400 BA Assen, The Netherlands. This committee is acknowledged by the Central Committee on Research Involving Human Subjects (known by its Dutch initials, CCMO). Patients and healthy controls were recruited from the local region. All participants signed an informed consent form.

### Description of Healthy Controls

In total, 209 age- and gender-matched healthy volunteers (age range 20–73 years), 138 females (age 45.3±13.4 years) and 71 males (age 48.7±14.0 years) underwent the QST assessments on their dorsal hand and foot. These body locations have been indicated by Rolke et al., 2006 as reference sites for QST [6]. A previous study concluded that there were no significant differences in QST parameters between the right and left sides of the body in healthy volunteers [6], thus we obtained QST reference values from one side of the body. In total, 418 QST references from the upper and the lower extremity were obtained. Healthy volunteers were identified according to medical history. Subjects were specifically questioned about previous injuries or diseases. The healthy subjects did not use pain medication regularly and were free of medication at the time of the assessments.

### Description of the Patient Cohort

Patients were recruited from the outpatient Department of the Pain Management Unit of the University Medical Center Groningen, The Netherlands. All patients were diagnosed as suffering from neuropathic pain by the physicians of the pain management unit. Neuropathic pain diagnosis was made on grounds of coherent patient history, medical history, physical examination, including neurologic function tests such as EMG. Each clinical diagnosis was additionally confirmed by an experienced pain specialist of the Pain Management Unit based on patient’s files. In total, 81 neuropathic pain patients (43 females age 52.6±12.7 years and 38 males age 49.8±13.0 years) underwent the QST assessment, each at the area where the most profound pain was experienced and at their contralateral counterpart (leg: n = 42, arm: n = 19, thorax: n = 7, groin: n = 4, shoulder: n = 3, back: n = 2, neck: n = 1, abdomen: n = 1, flank: n = 1).

Prior to undergoing the QST assessments, patients were asked to rate their ongoing pain level using a Numerical Rating Scale (NRS) of ‘0’ indicating ‘‘no pain’’, and ‘100’ indicating ‘‘most intense pain imaginable’’. Patients did not discontinue their regular pain treatment if applicable.

### Quantitative Sensory Testing (QST)

The QST battery consisted of seven tests, measuring thirteen parameters and was applied according to the standardized protocol of Rolke et al., 2006 [6]. QST was performed by two research nurses, who underwent a comprehensive training at the DNFS in Germany. All tests were performed at the same research facility of PRA Int., Groningen, The Netherlands. The average room temperature was 22.8°C; SD ±1.8°C.

Thermal QST tests were performed using the Medoc Pathway System (Medoc, Israel) and consisted of six parameters: threshold assessments for warm and cold detection (WDT, CDT) and heat pain and cold pain (HPT, CPT). In addition, paradoxical heat sensations (PHS) during the thermal sensory limen (TSL) procedure of alternating warm and cold stimuli were identified.

Mechanical QST tests consisted of seven different parameters. The mechanical detection threshold (MDT) was determined with a standardized set of modified von Frey filaments (Optihair2-Set, Marstock Nervtest, Germany). The mechanical pain threshold (MPT) was measured using a set of seven pinprick devices (flat contact area of 0.2 mm in diameter) with fixed stimulus intensities that exerted forces of 8, 16, 32, 64, 128, 256, and 512 mN. Mechanical pain sensitivity (MPS) was assessed using the same set of seven weighted pinprick stimuli to obtain a stimulus–response function for pinprick-evoked pain. Dynamic mechanical allodynia (DMA) was assessed as part of the test above, using a set of three light tactile stimulators as dynamic innocuous stimuli: cotton wisp, cotton wool tip fixed to an elastic strip and a standardized brush (SENSElab No.5, Somedic, Sweden).

Vibration detection threshold (VDT) was performed with a Rydel–Seiffer graded tuning fork (64 Hz, 8/8 scale) that was placed over a bony prominence. The wind up ratio (WUR) test was assessed with a pinprick intensity of 256 mN. The pressure pain threshold (PPT) was determined over muscle with a pressure gauge device (FDN200, Wagner Instruments, CT, USA).

### Calibration of QST Equipment

The Medoc Pathway System was maintained and calibrated according to the manufactures guideline (calibration took place every three months, cleaning of the system every 6 months). All mechanical devices were inspected regarding their function prior to each testing. The standardized von Frey filaments (Marstock) were replaced once a filament of concern was bent. Each pinprick device underwent a functionality test and inspection of the contact tip. The tip was replaced once bent including a calibration of the device. Two of the three light tactile stimulators i.e. cotton wisp and cotton wool tip fixed to an elastic strip were replaced after each testing. The standardized brush was replaced once the brush hairs were not conforming to the usual shape (typically every 6 months). The pressure gauge device and tuning fork were not replaced during the assessment period.

### Z-transformation of QST Data

QST data of patients with neuropathic pain were compared with reference data from gender and age matched healthy volunteers. Both, patients and healthy subjects were divided into three age groups each (20–45 years of age, 46–60 years of age and 61–75 years of age). QST values of chronic pain locations and their mirror image area at the upper extremities were compared to QST reference values obtained from the dorsal hand of healthy controls (n = 63 for females and n = 29 for males for age group 20–45 years; n = 58 for females and n = 24 for males for age group 46–60 years; n = 17 for females and n = 18 for males for age group 61–75), whereas values from chronic pain locations at lower extremities and their mirror image area were compared to reference values obtained from the dorsal foot of healthy controls (n = 63 for females and n = 29 for males for age group 20–45 years; n = 58 for females and n = 24 for males for age group 46–60 years; n = 17 for females and n = 18 for males for age group 61–75). QST values from each patient were transformed to z-scores as described by Rolke et al., 2006 [6]. A score above 1.96 or below −1.96 falls outside the 95% confidence interval of the mean reference value and was considered as a sensory abnormality. Abnormalities were subsequently categorized as either a sensory gain or a sensory loss.

Because “dynamic mechanical allodynia” (DMA) never occurs in healthy volunteers, the QST parameter could not be used for z-score analysis. Alternatively, patients ratings greater than NRS 10 (scale 0–100) were regarded as clinically relevant and were identified as abnormal.

For the QST parameter “wind up ratio” (WUR), twenty-three patients (thirteen assessments at the affected side and ten assessments at the contralateral side) rated the single pinprick stimulus as “0” making ratio calculations (painfulness of one pinprick stimulation vs. painfulness of a train of ten pinprick stimulations) for Wind-up impossible. For these patients WUR was not used for subsequent analyses.

### Proportion of Patients with Sensory Abnormalities at the Affected Side

For each QST parameter, the proportion of patients with sensory abnormalities at the painful, affected side was calculated. To estimate the prevalence of sensory abnormalities in the general patient population we calculated the 95% confidence intervals of the calculated proportions using the ‘Wilson Estimate’ of proportion [17]. These 95% confidence intervals give an indication of the expected range of the occurrence of abnormalities in the general pain patient population with neuropathic pain and tests whether the proportion differs significantly from zero (p<0.05).

### Proportion of Patients with Sensory Abnormalities at the Contralateral Side

For each QST parameter, the proportion of patients with sensory abnormalities at the non-painful, contralateral side was calculated applying the same procedure (see 2.4.2.).

### Proportion of Patients with Sensory Abnormalities for the Same QST Parameter at the Affected and Contralateral Side

For each patient, the presence or absence of a sensory abnormality at the contralateral side for a particular QST parameter was determined when the patient had already shown a sensory abnormality for this QST parameter at the affected side. This allowed the direct identification of a relationship between bilateral sensory abnormalities for the same QST parameter. To increase statistical power we recalculated the above proportions but now pooled the thermal QST parameters (CPT, HPT, WDT, CDT, TSL and PHS) into one overall thermal QST domain and pooled the mechanical QST parameters (WUR, MPT, MPS, MDT, VDT, PPT and ALL) into one overall mechanical domain.

Again we estimated the prevalence of sensory abnormalities in the general patient population with the ‘Wilson Estimate’. All proportions are reported as percentages.

### Correlation between Background Pain and Sensory Abnormalities

To identify correlations between ongoing background pain and values for each QST parameter Pearson correlations were calculated.

### Correlation between Numbers of Sensory Abnormalities at the Affected and Contralateral Side

The overall numbers of sensory abnormalities for the affected and contralateral side across the thirteen QST parameters were compared to identify possible relationships using Pearson correlations.

## Results

### QST Observations in Healthy Controls

From the healthy volunteer cohort (n = 209) investigated in this study, a total of 418 locations were assessed and 5434 measurements were analysed by means of z-score profiling.

### Sensory Function in Healthy Controls

Although the majority of the QST results obtained in healthy volunteers confirmed normal sensory function for this cohort, incidental sensory abnormalities (4.3%) were observed for all QST parameters with the exception of DMA. Age, -gender, -and location matched normative QST data are presented in Table S1.

**Figure 1:**
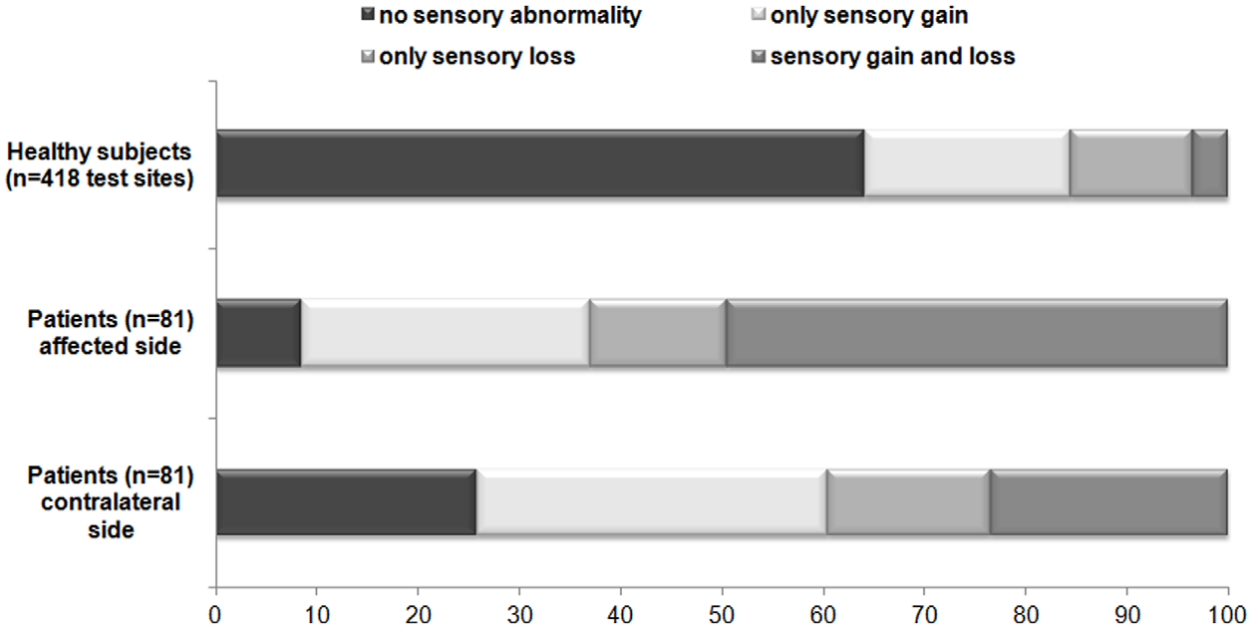
Sensory findings in healthy controls and neuropathic pain patients Sensory findings (gain and/or loss of sensory function) in % for healthy controls (n = 208 with 418 test sides) and for patients at the affected and contralateral side (n = 81). Sensory abnormalities were defined as Z score <−1.96 or >1.96 corresponding with 95% of values obtained from healthy volunteers. “No sensory abnormalities”: none of the Quantitative Sensory Testing (QST) parameters were outside the 95% CI. “Only sensory gain”: at least one QST parameter indicating thermal or mechanical hyperesthesia or hyperalgesia without the presence of hypoesthesia or hypoalgesia. “Only sensory loss”: at least one QST parameter indicating thermal or mechanical hypoesthesia or hypoalgesia without the presence of hyperesthesia or hyperalgesia. “Sensory gain and loss”: at least one positive sign combined with one negative sign.

Out of the total of 418 different body areas that were tested across all healthy controls 64.0% (258 locations) showed normal sensory function and 36.0% (160 locations) showed a sensory abnormality for at least one QST parameter. Sensory abnormalities were regarded as sensory gain in 20.3%, sensory loss in 12.0% and a mixture of sensory gain and sensory loss in 3.6% of the cases (Fig. 1).

### Demographics of Patients

Demographic data of the patients are shown in Table 1. All patients reported ongoing spontaneous pain only at their affected side ranging from 3 to 90 (Mean 64.1±21.4 SD) on a 0–100 NRS just before the QST assessment took place. The aetiology of patient’s pain in our sample was quite diverse, but did not include central pain patients. The largest subgroups developed pain after a surgical intervention (n = 27) including one patient with Complex Regional Pain Syndrome-II (CRPS) followed by an accident with trauma including fractures (n = 26). Other patients reported pain after failed back surgery (n = 8), Herniated nucleus pulposus (n = 7), amputation (n = 4), Radiotherapy (n = 3), peripheral nerve entrapment (n = 2). Three patients were diagnosed with postherpetic neuralgia and one patient was with Meralgia paresthetica (see Table 1).

**Table 1:** Patient characteristics.

ID	Gender	Age	NRS	Cause of Pain	Nerve	Clinical diagnosis	A.-side	C.- side
1	F	25	70	Accident with trauma	N. digitalis	peripheral nerve injury	3	2
2	F	39	80	Postsurgical pain	N. radialis	peripheral nerve injury	1	1
3	F	41	40	Postsurgical pain	TH 11	peripheral nerve injury	3	0
4	F	41	70	Metacarpal fracture	N. ulnaris	peripheral nerve injury	3	0
5	F	46	75	Accident with trauma	C 6	peripheral nerve injury	1	1
6	F	46	85	Postsurgical pain	N. digitalis palmaris	peripheral nerve injury	2	2
7	F	46	40	Amputation	N. cutaneous brachii	peripheral nerve injury	3	1
8	F	48	60	Accident with trauma	N. ulnaris	peripheral nerve injury	0	0
9	F	51	80	Peripheral nerve entrapment	C 6/7	peripheral nerve injury	4	2
10	F	51	70	Postsurgical pain	TH 11/12	peripheral nerve injury	2	2
11	F	53	80	Radiotherapy	TH 3–TH 6	peripheral nerve injury	4	3
12	F	64	60	Accident with trauma	TH 9/10	peripheral nerve injury	3	1
13	F	66	75	Accident with trauma	Cranial nerve XI	peripheral nerve injury	3	2
14	F	67	85	Herniated nucleus pulposus	TH 6/7	peripheral nerve injury	3	0
15	F	71	3	Herpes zoster	TH 12	postherpetic neuralgia	6	1
16	F	73	25	Herpes zoster	TH 11	postherpetic neuralgia	3	0
17	F	27	70	Femur fracture	N. sapheneus internus	peripheral nerve injury	8	2
18	F	36	80	Cruris fracture	N. tibialis	peripheral nerve injury	6	3
19	F	37	80	Postsurgical pain	TH 9/10	peripheral nerve injury	2	3
20	F	40	70	Amputation	N. tibialis	peripheral nerve injury	4	1
21	F	41	70	Postsurgical pain	N. peroneus/N. tibialis	peripheral nerve injury	4	0
22	F	42	70	Herniated nucleus pulposus	N. peroneus prof.	peripheral nerve injury	4	1
23	F	43	75	Accident with trauma	N. tibialis	peripheral nerve injury	4	2
24	F	43	75	Meralgia paresthetica	N. femoralis	peripheral nerve injury	2	3
25	F	46	75	Accident with trauma	N. peroneal	peripheral nerve injury	3	2
26	F	47	80	Accident with trauma	L 4	peripheral nerve injury	2	3
27	F	49	50	Accident with trauma	N. tibialis	peripheral nerve injury	4	5
28	F	49	30	Failed back surgery	L 5-S 1	peripheral nerve injury	5	3
29	F	50	10	Radiotherapy	Plexus brachialis	peripheral nerve injury	3	3
30	F	52	100	Failed back surgery	L 5/6	peripheral nerve injury	3	2
31	F	55	90	Herniated nucleus pulposus	L 5-S 1	peripheral nerve injury	3	2
32	F	56	90	Postsurgical pain	N. Suralis	peripheral nerve injury	2	1
33	F	58	90	Postsurgical pain	N. femoralis	peripheral nerve injury	2	1
34	F	59	60	Postsurgical pain	N. plantaris	peripheral nerve injury	5	1
35	F	61	80	Postsurgical pain	L 4/5	peripheral nerve injury	2	3
36	F	62	80	Failed back surgery	L 5-S 1	peripheral nerve injury	6	0
37	F	65	70	Herniated nucleus pulposus	L 5-S 1	peripheral nerve injury	5	6
38	F	65	50	Amputation	Peroneal nerves	peripheral nerve injury	7	1
39	F	66	70	Postsurgical pain	N. peroneus	peripheral nerve injury	2	1
40	F	66	90	Postsurgical pain	N. tibialis	peripheral nerve injury	5	3
41	F	71	65	Failed back surgery	L 5-S 1	peripheral nerve injury	3	2
42	F	72	80	Failed back surgery	L 4/5	peripheral nerve injury	2	2
43	F	75	80	Herniated nucleus pulposus	L 4/5	peripheral nerve injury	4	2
44	M	23	70	Postsurgical pain	TH 8/9	peripheral nerve injury	2	2
45	M	26	85	Accident with trauma	C 8	peripheral nerve injury	5	0
46	M	32	40	Postsurgical pain	TH 11	peripheral nerve injury	4	1
47	M	38	90	Accident with trauma	N. brachialis	peripheral nerve injury	1	6
48	M	47	80	Accident with trauma	L 4/5	peripheral nerve injury	8	5
49	M	42	70	Failed back surgery	L 5/6	peripheral nerve injury	7	2
50	M	43	90	Postsurgical pain	TH 10/12	peripheral nerve injury	6	3
51	M	49	40	Accident with trauma	N. ulnaris	peripheral nerve injury	2	1
52	M	50	70	Radiotherapy	TH 2	peripheral nerve injury	1	4
53	M	50	75	Rib fracture	TH 11	peripheral nerve injury	6	2
54	M	52	55	Postsurgical pain	N. ulnaris	CRPSII	2	2
55	M	53	45	Accident with trauma	N. ulnaris	peripheral nerve injury	2	2
56	M	55	50	Accident with trauma	N. radialis	peripheral nerve injury	2	1
57	M	56	55	Postsurgical pain	N. axillaris	peripheral nerve injury	1	1
58	M	58	40	Herniated nucleus pulposus	C 5-C 7	peripheral nerve injury	0	0
59	M	58	80	Postsurgical pain	N. radialis	peripheral nerve injury	1	1
60	M	59	60	Accident with trauma	N. radialis	peripheral nerve injury	2	1
61	M	60	80	Postsurgical pain	C 4	peripheral nerve injury	0	1
62	M	63	65	Accident with trauma	N. digiti	peripheral nerve injury	0	0
63	M	73	10	Herpes zoster	TH 8	postherpetic neuralgia	0	0
64	M	24	50	Postsurgical pain	N. Ilioinguinalis	peripheral nerve injury	8	2
65	M	28	75	Postsurgical pain	N. tibialis	peripheral nerve injury	5	1
66	M	40	60	Amputation	N. plantaris	peripheral nerve injury	2	1
67	M	41	3	Failed back surgery	S 1	peripheral nerve injury	4	1
68	M	43	60	Accident with trauma	L 4	peripheral nerve injury	4	1
69	M	44	40	Accident with trauma	N. femoralis	peripheral nerve injury	1	0
70	M	44	35	Postsurgical pain	N. Ilioinguinalis	peripheral nerve injury	2	0
71	M	46	65	Failed back surgery	L 4/5	peripheral nerve injury	0	0
72	M	47	75	Postsurgical pain	N. tibialis	peripheral nerve injury	4	1
73	M	51	70	Postsurgical pain	N. femoralis	peripheral nerve injury	3	0
74	M	53	70	Accident with trauma	L 5	peripheral nerve injury	0	0
75	M	54	50	Postsurgical pain	N. grenito-femoralis	peripheral nerve injury	2	0
76	M	57	75	Tibia fracture	N. tibialis	peripheral nerve injury	5	4
77	M	62	70	Accident with trauma	N. peroneus	peripheral nerve injury	2	0
78	M	63	80	Postsurgical pain	N. saphenus	peripheral nerve injury	1	1
79	M	65	65	Herniated nucleus pulposus	L 5	peripheral nerve injury	5	4
80	M	73	10	Peripheral nerve entrapment	L 5-S 1	peripheral nerve injury	3	2
81	M	75	60	Postsurgical pain	N. tibialis	peripheral nerve injury	1	2

Demographic patient overview; Patient ID, gender and age are indicated. Patient’s rating of ongoing pain prior to Quantitative Sensory Testing (QST) using a Numeric Rating scale (NRS) indicating “0” as “no pain” and “100” as the ‘‘most intense pain imaginable’’. Involved nerve indicates nerves (N.) or innervations area of nerves affected in relation to the cause of pain. Number of QST abnormalities refers to parameter exceeding CI 95% of z-scores (<−1.96 or >1.96) of values obtained from healthy volunteers for the affected (A.) and contralateral (C.) side of patients.

### QST Observations in Patients

For the 81 patients investigated in this study, 2106 QST data measurements were obtained from both the affected and contralateral side. The total of 2083 measurements were analysed by means of z-score profiling.

### Sensory Function in Patients

**Figure 2:**
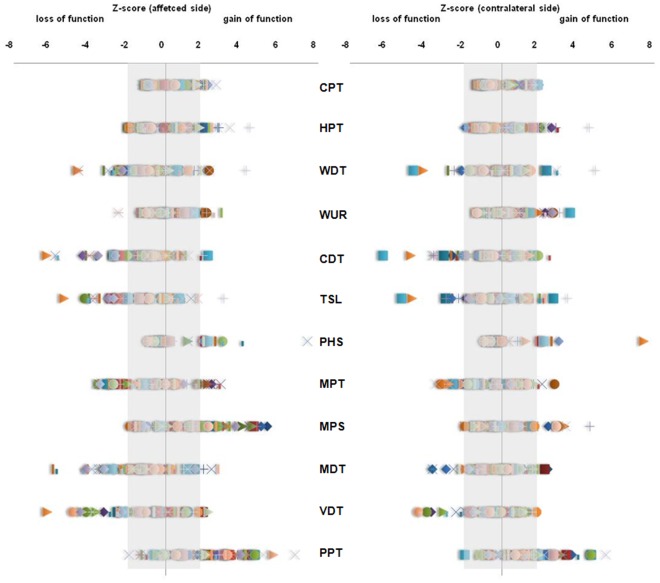
Z-score profiles at the affected side and contralateral side in neuropathic pain patients. Sensory abnormalities (Z score <−1.96 or >1.96) for each Quantitative Sensory Testing (QST) parameter at the affected side (left) and contralateral side (right) in 81 neuropathic pain patients. Grey area indicates parameters within the normal range (−1.96<Z<1.96 corresponding with 95% of values obtained from healthy volunteers). QST parameters: Cold Pain Threshold (CPT), Heat Pain Threshold (HPT), Warm Detection Threshold (WDT), Wind Up Ratio (WUR), Cold Detection Threshold (CDT), Thermal Sensory Limen (TSL), Paradoxical Heat Sensation (PHS), Mechanical Pain Threshold (MPT), Mechanical Pain Sensitivity (MPS), Mechanical Detection Threshold (MDT), Vibration Disappearance Threshold (VDT), Pressure Pain Threshold (PPT).

In patients with neuropathic pain, sensory abnormalities were observed in all QST parameters at both affected and contralateral side (Fig. 2). In our patient cohort, 91% had at least one QST abnormality at the affected side. Of the patients without sensory abnormalities at the affected side (9%), 28% still showed at least one sensory abnormality at the contralateral side. At the affected side, 49% of the patients had a mixture of sensory gain and loss, 28% had only sensory gain (hyperalgesia), and 14% had only sensory loss (hypoesthesia) (Fig. 1).

At the contralateral side, 74% of the patients had at least one QST abnormality. In 24% of the patients a mixture of sensory gain and loss was present. Almost 35% of the patients showed only sensory gain and 16% had only sensory loss at the contralateral side (Fig. 1).

95% Confidence Intervals confirmed that the prevalence of normal sensory function differs significantly between healthy controls and patients at the painful and non-painful side (all p<0.05). A significant difference was also present between the painful side and non-painful side of the patients (p<0.05).

### Sensory Changes at Patients Affected Side

Sensory abnormalities at the affected side ranged from 8.6% (n = 7) for CPT to 49.4% (n = 40) for PPT. 95% Confidence Intervals confirmed that the prevalence differed significantly from zero (p<0.05) for all QST parameters with highest incidence for MPT (95CI: 27%-48%) and PPT (95CI: 39%-60%) (Table 2A).

**Table 2:** Overview of sensory abnormalities in QST.

2A													
QST parameter affected side	CPT	HPT	WDT	WUR	CDT	TSL	PHS	MPT	MPS	MDT	VDT	PPT	DMA
n of sensory abnormality	7	9	18	6	23	24	22	30	20	25	23	40	16
n of sensory gain	7	9	3	5	1	1	22	7	20	3	1	40	16
n of sensory loss	0	0	15	1	22	23	0	23	0	22	22	0	0
% of sensory abnormality	8.6	11.1	22.2	8.8	28.4	29.6	27.2	37.0	24.7	30.9	28.4	49.4	19.8
Wilson estimates lower CI 95%	4.0	5.8	14.5	3.9	19.7	20.8	18.7	27.3	16.6	21.9	19.7	38.8	12.5
Wilson estimates upper CI 95%	17.1	20.1	32.5	18.4	39.1	40.4	37.8	47.9	35.2	41.7	39.1	60.0	29.9
p<0.05	*	*	*	*	*	*	*	*	*	*	*	*	*
**2B**													
**QST parameter contralateral side**	**CPT**	**HPT**	**WDT**	**WUR**	**CDT**	**TSL**	**PHS**	**MPT**	**MPS**	**MDT**	**VDT**	**PPT**	**DMA**
n of sensory abnormality	0	9	12	7	13	13	8	14	10	8	8	25	3
n of sensory gain	0	9	6	7	1	6	8	2	8	2	0	25	0
n of sensory loss	0	0	6	0	12	7	0	12	2	6	8	0	0
% of sensory abnormality	0.0	11.1	14.8	9.9	16.0	16.0	9.9	17.3	12.3	9.9	9.9	30.9	3.7
Wilson estimates lower CI 95%	−0.9	5.8	8.6	4.6	9.5	9.5	4.9	10.5	6.7	4.9	4.9	21.9	0.9
Wilson estimates upper CI 95%	5.6	20.1	24.4	19.4	25.8	25.8	18.6	27.1	21.5	18.6	18.6	41.7	10.9
p<0.05	n.s.	*	*	*	*	*	*	*	*	*	*	*	*

Patient numbers with sensory abnormalities at the affected (Table 2A, top) and contralateral side (Table 2B, bottom). Sensory abnormalities were defined as Z score <−.96 or >1.96 corresponding with 95% of values obtained from healthy volunteers. Shown are direction (n of sensory gain/n of sensory loss) and overall abnormalities in percent (% of sensory abnormality) for each Quantitative Sensory Testing (QST) parameters in 81 chronic pain patients. QST parameter: Cold Pain Threshold (CPT), Heat Pain Threshold (HPT), Warm Detection Threshold (WDT), Wind Up Ratio (WUR), Cold Detection Threshold (CDT), Thermal Sensory Limen (TSL), Paradoxical Heat Sensation (PHS), Mechanical Pain Threshold (MPT), Mechanical Pain Sensitivity (MPS), Mechanical Detection Threshold (MDT), Vibration Disappearance Threshold (VDT), Pressure Pain Threshold (PPT) and Dynamic Mechanical Allodynia (DMA). Wilson estimates with upper and lower bound of the 95% CI for each QST parameter (* p<0.05).

**Figure 3:**
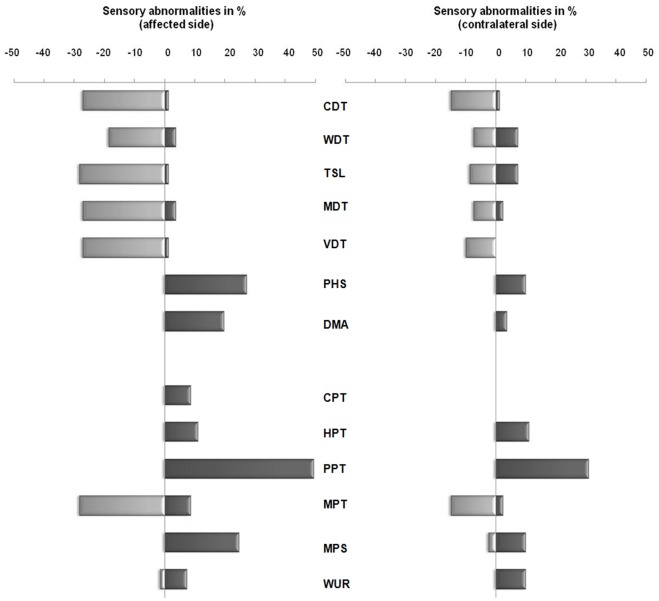
Quantitative Sensory Testing (QST) abnormalities at the affected and contralateral side in neuropathic pain patients. Quantitative Sensory Testing (QST) Z-score abnormalities in % at the affected (left) and contralateral side (right) in 81 neuropathic pain patients. Sensory abnormalities were defined as Z score < −1.96 or >1.96 corresponding with 95% of values obtained from healthy volunteers. QST parameter are ordered as sensory parameters: Cold Detection Threshold (CDT), Warm Detection Threshold (WDT), Thermal Sensory Limen (TSL), Mechanical Detection Threshold (MDT), Vibration Disappearance Threshold (VDT), Paradoxical Heat Sensation (PHS), Dynamic Mechanical Allodynia (DMA) and nociceptive parameters: Cold Pain Threshold (CPT), Heat Pain Threshold (HPT), Pressure Pain Threshold (PPT), Mechanical Pain Threshold (MPT), Mechanical Pain Sensitivity (MPS) and Wind Up Ratio (WUR). Z-scores with positive sensory signs (gain of sensory function) plotted rightwards and negative sensory signs (loss of sensory function) plotted leftwards. Absence of DMA is normal and therefore no negative sign possible.

For the nociceptive parameters (CPT, HPT, PPT, MPS, WUR) there were predominantly changes reflecting hyperalgesia, whereas for the non-nociceptive ones (CDT, WDT, TSL, MDT, VDT) they reflected hypoesthesia (Fig. 3).

For the nociceptive parameters CPT and HPT, thermal pain threshold were decreased indicating a thermal hyperalgesia. An increased pain due to blunt pressure (PPT) and an increased sensitivity to mechanical pain (MPS) were observed indicating only hyperalgesia for these parameters. For MPT a greater incidence for mechanical hypo- than hypersensitivity was detected. WUR was more frequently increased than decreased indicating a greater incidence for hyper- than hyposensitivity.

Thermal hypoesthesias were observed also in most of the patients for CDT, WDT and TSL. For MDT there was predominantly a sensory loss observed indicating a mechanical hypoesthesia. It was possible to detect hyperesthesia for VDT for one patient, but for the large majority VDT responses indicated hypoesthesia.

In 27% (n = 22) of the patients a sensory gain for PHS was detected at the affected side. DMA was present in 26% of the patients, in 6% of very mild intensity, however, 20% of patients showed a clinically relevant increased response for DMA indicating a dynamic allodynia.

### Sensory Changes at the Patient’s Contralateral Side

Sensory abnormalities at the contralateral side ranged from no abnormalities for CPT to 30.9% (n = 25) for PPT. With the exception of CPT, 95% Confidence Intervals confirmed that the prevalence differed significantly from zero (p<0.05) for all QST parameters with highest incidence for MPT (95CI: 11%-27%) and PPT (95CI: 22%-42%) (Table 2B).

Overall there were less sensory abnormalities at the contralateral side than at the affected side.

For nociceptive parameters there was predominantly sensory gain observed, suggesting the presence of hyperalgesia, whereas for non-nociceptive parameters predominantly a sensory loss was identified suggesting hypoesthesia (Fig. 3).

Only sensory gain for HPT, PPT and WUR were observed suggesting hyperalgesia. For MPT sensory loss was more frequently observed than sensory gain indicating a greater incidence for mechanical hyposensitivity than hypersensitivity. In contrast, for MPS was sensory gain was more frequently observed than sensory loss indicating a greater incidence for mechanical hypersensitivity than hyposensitivity.

Thermal hypoesthesias were observed for most of the cases for CDT and for TSL, only 7.4% of patients showed hyperesthesia for TSL and 1.2% for CDT. WDT abnormalities were observed in 14.8% of the cases and this was due to both sensory loss and gain. For MDT at the contralateral side sensory loss was observed three times as often as sensory gain, indicating a greater incidence for mechanical hypoesthesia. There was only hypoesthesia for VDT at the contralateral side.

In 9.8% of the patients a sensory gain for PHS at the contralateral side was detected. DMA was present in 19.8% of patients, but mostly of very mild intensity. However, 3.7% of patients showed a clinically relevant sensory gain for DMA indicating a dynamic allodynia.

### Example of Magnitude of Somatosensory Abnormalities

Here we describe one patient in greater detail for better understanding of the magnitude of somatosensory abnormalities based on raw values of the QST battery.

A 25 year old woman (ID1) suffered from a cut injury at her left hand with a lesion of the digitalis nerve. Subsequently, she developed severe pain at left palm including digits IV and V. Bedside tests using von Frey filaments and a brush confirmed impaired sensibility including allodynia of left hand. These sensory signs were within neuroanatomical plausible distribution of the digitalis nerve. The clinical diagnosis “peripheral nerve injury” was made. The QST assessment took place in the area of greatest pain complaints and on the same contralateral site. Normative data obtained from dorsal hand were from 63 age- and gender matched volunteer’s ± SD indicated in brackets. For the affected side the patient rated the different pinprick forces with a NRS score of 53.1 indicating an increased sensitivity for mechanical pain (MPS) (0.62±1.00). The NRS ratio for Wind up (WUR) test was increased to 6.5 suggesting central sensitisation (NRS 2.53±2.33). Her ratings for DMA pain of NRS 54.7 indicated allodynia. Clinically, this QST profile indicates a predominant gain of sensory function due to small and large fibre sensitisation. At the contralateral side the patient displayed a decreased threshold for MDT of 0.5 mN (2.22±2.27 mN) and a decreased threshold for CDT of 24.9°C (30.7±0.77°C). DMA pain of NRS 3.5 indicates minor allodynia. Clinically, for the contralateral side a predominant gain of sensory function was found indicating small and large fibre sensitisation.

### Sensory Changes at the Contralateral Side in Relation to Sensory Changes at the Affected Side

**Figure 4:**
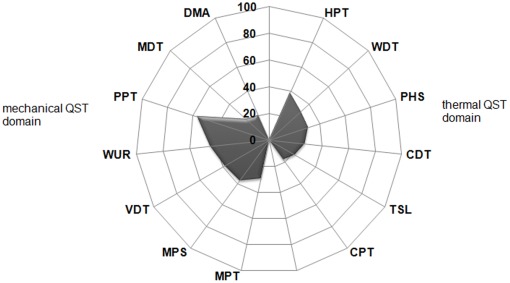
Incidence of QST abnormalities at the contralateral side in neuropathic pain patients. Incidence of QST abnormalities at the contralateral side in 81 neuropathic pain patients. Sensory abnormality in percent (%) of either gain or loss of function for each Quantitative Sensory Testing (QST) parameter at the contralateral side once there was already an abnormality detected for the same parameter at the affected side. Sensory abnormalities were defined as Z score < −1.96 or >1.96 corresponding with 95% of values obtained from healthy volunteers. QST parameter in this radar diagram are ordered as mechanical stimuli consisting of Mechanical Pain Threshold (MPT), Dynamic Mechanical Allodynia (DMA), Pressure Pain Threshold (PPT), Vibration Disappearance Threshold (VDT), Mechanical Detection Threshold (MDT), Mechanical Pain Sensitivity (MPS) and Wind Up Ratio (WUR) (left side) and thermal stimuli consisting of Cold Pain Threshold (CPT), Heat Pain Threshold (HPT), Warm Detection Threshold (WDT), Cold Detection Threshold (CDT), Thermal Sensory Limen (TSL), Paradoxical Heat Sensation (PHS) (right side).

To further investigate the extent of contralateral abnormalities we determined the presence or absence of abnormalities in each of the QST parameters at the contralateral side given that an abnormality for the same parameter was present at the affected side (Table 3). This occurred in 20% (for DMA) to 57% (for PPT) of the cases (Fig. 4). Confidence Intervals (95%) confirmed the prevalence to be significantly (p<0.05) different from zero for all QST parameters, with the exemption of CPT (see Table 3). The highest proportions were seen for the VDT (95CI: 20%-57%) and PPT (95CI: 42%-71%). Although all proportions were significant, some confidence intervals were very large due to small numbers of observations. This was especially true for WUR and for most of the thermal QST parameters.

**Table 3:** Overview of sensory abnormalities at the contralateral side given an abnormality at the affected side.

QST parameter	CPT	HPT	WDT	WUR	CDT	TSL	PHS	MPT	MPS	MDT	VDT	PPT	DMA
n of sensory gain similar to affected side	0	3	2	2	0	1	6	1	7	0	0	23	2
n of sensory loss similar to affected side	0	0	3	0	5	3	0	7	0	4	8	0	0
% of sensory abnormality similar to affected side	0.0	38.5	31.8	44.4	26.9	22.2	30.8	29.4	37.5	23.1	38.5	56.8	20.0
Wilson estimates lower CI 95%	−4.6	12.0	12.4	12.0	9.9	6.8	13.0	14.1	18.1	6.9	19.8	42.2	2.5
Wilson estimates upper CI 95%	41.0	64.9	51.3	76.9	44.0	37.6	48.5	44.7	56.9	39.3	57.2	71.5	37.5
p<0.05	n.s.	*	*	*	*	*	*	*	*	*	*	*	*

Patient numbers with sensory abnormalities and in their direction (n of sensory gain/n of sensory loss). Sensory abnormalities were defined as Z score < −1.96 or >1.96 corresponding with 95% of values obtained from healthy volunteers. Percent (% of sensory abnormality) indicates overall occurrence of sensory abnormalities for each Quantitative Sensory Testing (QST) parameter at the contralateral side once there was already an abnormality for the same parameter detected at the affected side in 81 chronic pain patients. QST parameters: Cold Pain Threshold (CPT), Heat Pain Threshold (HPT), Warm Detection Threshold (WDT), Wind Up Ratio (WUR), Cold Detection Threshold (CDT), Thermal Sensory Limen (TSL), Paradoxical Heat Sensation (PHS), Mechanical Pain Threshold (MPT), Mechanical Pain Sensitivity (MPS), Mechanical Detection Threshold (MDT), Vibration Disappearance Threshold (VDT), Pressure Pain Threshold (PPT) and Dynamic Mechanical Allodynia (DMA). Wilson estimates with upper and lower bound of the 95% CI for each QST parameter (* p<0.05).

To increase statistical power with the purpose to allow a more accurate estimation of the prevalence of sensory abnormalities in the general chronic pain patient population, thermal and mechanical QST parameters were combined into one thermal and one mechanical domain. For the affected side this grouping resulted in 21.4% (95CI: 18%–25%) thermal abnormalities and 29.0% (95CI: 25%–33%) mechanical abnormalities. For the contralateral side 11.6% (95CI: 9%–14%) thermal abnormalities and 13.7% (95CI: 11%–17%) mechanical abnormalities were found. To investigate the occurrence of bilateral manifestations of sensory abnormalities we calculated the prevalence of thermal and mechanical abnormalities at the contralateral side given that there was an abnormality at the affected side for the same QST domain in the same patient. This resulted in 95% CI’s for bilateral abnormality ranging from 12%–25% and 33%–50% for thermal and mechanical QST domains, respectively.

### Correlation between Background Pain and QST Parameters

All patients reported ongoing spontaneous pain (NRS mean 64.1, SD ±21.4) at their affected side before the QST assessment took place (see Table 1). There were no significant correlations found using Pearson correlations between background pain and QST parameters. A significant correlation was found between the frequencies of sensory abnormalities at the contralateral side with background pain (r = 0.221; p<0.05). Furthermore, this effect was supported by the correlation between the increase of sensory loss at the contralateral side and background pain (r = 0.236; p = 0.05).

### Correlation between Numbers of Sensory Abnormalities at the Affected and Contralateral Side

The number of sensory abnormalities for patients varied between 0 and 8 for the affected and 0 and 6 for the contralateral side for the thirteen QST parameters assessed (see Table 1). The overall occurrence of contralateral abnormalities were significantly correlated with abnormalities at the affected side (r = 0.290; p<0.01). Furthermore, a correlation was observed between the presence of sensory loss at the affected and contralateral side (r = 0.300; p<0.01), whereas for the presence of sensory gain at the affected and contralateral side a stronger correlation was found (r = 0.575; p<0.01).

## Discussion

This QST study shows that patients with unilateral neuropathic pain have a diversity of sensory abnormalities at the painful side, and to a lesser extent, at the contralateral non-painful side. Using the standardized QST protocol with 13 different parameters to obtain a complete sensory profile, it was demonstrated that bilateral sensory abnormalities are apparent in a considerable number of the patients that experience chronic unilateral pain.

There was a significant correlation between the number of abnormalities at the painful side and the contralateral side. Even more so, if a particular abnormality was detected at the painful side, this abnormality was then the most likely abnormality to occur contralaterally. This was particularly striking for the mechanical stimuli group where the estimated prevalence of sensory abnormalities at the non-painful side was 33%–50% (95% CI) once a mechanical abnormality was detected at the painful side. These results have implications for the evaluation of patients in clinical practice, since often the non-affected side is used as the reference side. Our results show that using the contralateral side as the reference to identify sensory abnormalities at the affected side might lead to misinterpretation of the clinical manifestation.

### Somatosensory Function in Healthy Controls

Z-score transformation of QST data revealed one or more somatosensory abnormalities in 36% of all members of the healthy control group. This number is in line with previous findings reporting 41% abnormalities using the QST protocol [16].

In our healthy volunteers, abnormalities were observed across all QST parameters with the exception of DMA. The detected sensory abnormalities reflected gain of function for the most part, some loss of function and in a minority fraction both gain and loss of function (Fig. 1).

Although previously reported otherwise [16], in the present study, PHS >1 occurred in 1.4% at the test side “dorsal hand” and in 9.3% at the test side “dorsal foot”. The presence of abnormal PHS in our study could be associated with a greater likelihood of sensory dysfunction with an increase in age (r = 0.193; p<0.01).

### Sensory Signs at the Affected, Painful Side of Neuropathic Pain Patients

As expected, the large majority (91%) of neuropathic pain patients showed sensory abnormalities at their affected side. Maier and colleagues (2010) [16] reported a similar percentage (92%) of patients with at least one QST abnormality. Given the fact that for 9% of the patients, no abnormality could be detected, QST and the cut-off of 95% CI of the mean reference values might possibly be more stringent than clinical examination.

In accordance with previous studies, sensory loss was predominantly found in non-nociceptive parameters [16], [18] which could be associated with central or peripheral neuronal damage which might lead to ongoing pain via increased ectopic activity [19], [20], [21]. Sensory gain was predominantly found in nociceptive parameters which could be associated with peripheral sensitization and/or altered central processing [10], [22], [23], [24], [25].

Maier and co-workers (2010) reported abnormal QST values for the affected side across the different clinical neuropathic pain entities ranging between 8%–36% (compared to 7%–48% in this study) [16]. There was good agreement between our estimates of the expected range of sensory abnormalities in the general neuropathic pain patient population and those reported by Maier [16]. Only estimate ranges for the occurrence of sensory abnormalities for CDT, WUR, TSL, MDT, MPT and VDT in the present study differed slightly but were still in close proximity to the values reported previously [16].

### Contralateral Sensory Signs in Neuropathic Pain Patients

Contralateral sensory changes in patients with chronic pain have been acknowledged in a number of clinical studies [3], [4], [5], [26], [27], [28], [29]. For instance it was reported that 5% of the patients with complex regional pain syndrome (CRPS) present bilateral symptoms [30]. Despite reports of the existence of bilateral changes in chronic pain patients, no elaborate quantitative data have been published. In the present QST study, sensory abnormalities at the contralateral side were observed for all QST parameters.

Comparing the overall sensory findings from the contralateral side with those obtained from the healthy controls, a significant difference was shown indicating abnormal sensory function at the contralateral side in patients with unilateral neuropathic pain. In addition, a significant correlation was found for abnormal sensory function at the contralateral sides compared to the affected side. The pattern of sensory abnormalities for nociceptive and non-nociceptive parameters at the contralateral side was in line with that at the affected side but less severe. All patients had unilateral pain causing events and most showed bilateral sensory abnormalities. This finding points to a central component in processing the pain and controlling sensory function bilaterally.

Preclinical studies have also found evidence for bilateral sensory changes upon unilateral induction of pain and these studies correlated the severity of pain with occurrence of bilateral changes. Hubbard and colleagues (2008) demonstrated in a rat model using painful cervical nerve root compression that the occurrence of contralateral allodynia depended on the load of compression [31]. In another study, zymosan induced sciatic neuritis in rats, causing a dose-dependent bilateral allodynia [32].

In healthy volunteers and patients with rheumatoid arthritis, an intradermal administration of capsaicin induced mechanical hyperalgesia and allodynia at the side contralateral to the injection area [29]. Studies using capsaicin revealed that short-lasting but high intensity pain induces contralateral sensory changes [7]; [21]. These results suggest that pain intensity may play a prominent role in contralateral sensory changes. Potential underlying conditions which may lead to contralateral sensory changes in unilateral pain condition are currently being investigated. Koltzenburg et al. [33] suggested the involvement of nerve growth factors (NGF) to explain the contralateral peripheral responses in rats with unilateral neural injuries. Other studies suggested the involvement of altered glial activation and spinal pro-inflammatory cytokines (tumour necrosis factor (TNF), interleukin-1 (IL-1), interleukin-6 (IL-6)) [34], [35], [36], [37].

In line with these findings, in the present study we have also found a significant correlation between background pain and QST abnormalities at the contralateral side in patients who experienced a unilateral pain-causing event. This supports previous suggestions that high pain intensities can induce sensory abnormalities at the contralateral side in patients with unilateral pain. Additional studies are needed to evaluate, for instance, if the severity of pain determines the onset of contralateral changes and if ongoing pain is the driving force for the maintenance of contralateral abnormalities in chronic pain patients.

**Table 4:** Diagnostic consequence of using either the contralateral side or normative data from healthy volunteers.

observation affected side	observation contralateral side	clinical result using contralateral side as reference	clinical result using healthy volunteers as reference
0	0	0	0
+	0	+	+
−	0	−	−
0	+	−	0
0	−	+	0
+	+	0	+
+	−	++	+
−	+	–	−
−	−	0	−

The interpretation of sensory function and its clinical manifestation at the affected side using the contralateral side or reference values from healthy volunteers. The observation at the affected side and contralateral side indicate the sensory response for a Quantitative Sensory Testing (QST) parameter in patients. Clinical results using the contralateral side as reference or healthy volunteers as reference indicate sensory interpretation for the affected side. ‘0’ indicate normal sensory function, ‘+’ indicate sensory gain such as hyperalgesia/allodynia, ‘−’ indicate sensory loss such as hypoesthesia, ‘++’ or ‘− −’ indicate overestimation of sensory gain or sensory loss, respectively.

### Correlation between Sensory Changes at Affected and Contralateral Side

An interesting finding is that the presence of sensory gain or loss at the affected side was related to sensory gain or loss for the same QST parameter at the contralateral side, ranging from 20% to 57% dependent on the QST parameter. In particular for the group of mechanical stimuli, the estimated presence of sensory abnormalities at the non-affected side was substantial (ranging from 33%–50%). Although sensory abnormalities at the contralateral side were less pronounced compared to those at the affected side, there was a significant correlation between the numbers of sensory abnormalities at both sides in patients.

Contrary to recommendations to use the contralateral side as a reference to identify sensory abnormalities in patients [1], [38] our data indicate that this is not advisable since the sensory function at the contralateral side stands a reasonable chance of being altered. The interpretation of sensory signs and subsequent clinical manifestations using the contralateral side or reference values from healthy volunteers may vary (see Table 4 for examples).

Since most (n = 77) patients in the present study continued their pain medication it cannot be excluded that the medication itself might have influenced the onset or maintenance of somatosensory changes seen bilaterally.

These results firmly establish evidence for a cautious use of the contralateral side as a reference site in clinical practice. A way to overcome this problem is the use of reference values for either normative response or for pathological response to QST parameters to allow a precise identification of sensory abnormalities in patients.

In conclusion, our data provide detailed evidence for bilateral sensory abnormalities in unilateral neuropathic pain patients using a standardized, elaborate QST protocol. Our results show that in these patients, the contralateral side should not be regarded as normal or healthy *per se*. This has implications for appropriate diagnostic evaluation in clinical practice.

## Supporting Information

Table S1 **Quantitative Sensory Testing normative data of healthy volunteers.** Quantitative Sensory Testing (QST) normative values assessed on dorsal hand (A) and dorsal foot (L) for female (F) and male (M) according to three age groups (18–45 years (y), 46–60 years and 61–75 years of age) in healthy volunteers. QST parameters: Cold Pain Threshold (CPT), Heat Pain Threshold (HPT), Warm Detection Threshold (WDT), Wind Up Ratio (WUR) (intensity of perception of single vs. series of 1 Hz stimuli as NRS), Cold Detection Threshold (CDT), Thermal Sensory Limen (TSL), Paradoxical Heat Sensation (PHS), Mechanical Pain Threshold (MPT), Mechanical Pain Sensitivity (MPS), Mechanical Detection Threshold (MDT), Vibration Disappearance Threshold (VDT), Pressure Pain Threshold (PPT). The absence of DMA is normal and therefore described. Numeric Rating scale (NRS) indicate “0” as “no pain” and “100” as the ‘‘most intense pain imaginable’’. Unit of each QST parameter is indicated. Values indicate mean ± SD for each QST parameter.
(TIF)

## References

[1] Haanpaa ML, Backonja MM, Bennett MI, Bouhassira D, Cruccu G (2009). Assessment of neuropathic pain in primary care.. Am J Med.

[2] Walk D, Sehgal N, Moeller-Bertram T, Edwards RR, Wasan A (2009). Quantitative sensory testing and mapping: a review of nonautomated quantitative methods for examination of the patient with neuropathic pain.. Clin J Pain.

[3] Huge V, Lauchart M, Forderreuther S, Kaufhold W, Valet M (2008). Interaction of hyperalgesia and sensory loss in complex regional pain syndrome type I (CRPS I).. PLoS One.

[4] de la Llave-Rincon AI, Fernandez-de-las-Penas C, Fernandez-Carnero J, Padua L, Arendt-Nielsen L (2009). Bilateral hand/wrist heat and cold hyperalgesia, but not hypoesthesia, in unilateral carpal tunnel syndrome.. Exp Brain Res.

[5] Fernandez-de-las-Penas C, de la Llave-Rincon AI, Fernandez-Carnero J, Cuadrado ML, Arendt-Nielsen L (2009). Bilateral widespread mechanical pain sensitivity in carpal tunnel syndrome: evidence of central processing in unilateral neuropathy.. Brain.

[6] Rolke R, Baron R, Maier C, Tolle TR, Treede RD (2006). Quantitative sensory testing in the German Research Network on Neuropathic Pain (DFNS): standardized protocol and reference values.. Pain.

[7] Rolke R, Magerl W, Campbell KA, Schalber C, Caspari S (2006). Quantitative sensory testing: a comprehensive protocol for clinical trials.. Eur J Pain.

[8] Hansson P, Backonja M, Bouhassira D (2007). Usefulness and limitations of quantitative sensory testing: clinical and research application in neuropathic pain states.. Pain.

[9] Shy ME, Frohman EM, So YT, Arezzo JC, Cornblath DR (2003). Quantitative sensory testing: report of the Therapeutics and Technology Assessment Subcommittee of the American Academy of Neurology.. Neurology.

[10] Treede RD, Meyer RA, Raja SN, Campbell JN (1992). Peripheral and central mechanisms of cutaneous hyperalgesia.. Prog Neurobiol.

[11] Treede RD, Rolke R, Andrews K, Magerl W (2002). Pain elicited by blunt pressure: neurobiological basis and clinical relevance.. Pain.

[12] Felix ER, Widerstrom-Noga EG (2009). Reliability and validity of quantitative sensory testing in persons with spinal cord injury and neuropathic pain.. J Rehabil Res Dev.

[13] Geber C, Klein T, Azad S, Birklein F, Gierthmuhlen J (2011). Test-retest and interobserver reliability of quantitative sensory testing according to the protocol of the German Research Network on Neuropathic Pain (DFNS): a multi-centre study.. Pain.

[14] Gierthmuhlen J, Maier C, Baron R, Tolle T, Treede RD (2011). Sensory signs in complex regional pain syndrome and peripheral nerve injury..

[15] Freynhagen R, Rolke R, Baron R, Tolle TR, Rutjes AK (2008). Pseudoradicular and radicular low-back pain–a disease continuum rather than different entities? Answers from quantitative sensory testing.. Pain.

[16] Maier C, Baron R, Tolle TR, Binder A, Birbaumer N (2010). Quantitative sensory testing in the German Research Network on Neuropathic Pain (DFNS): Somatosensory abnormalities in 1236 patients with different neuropathic pain syndromes..

[17] Moore DS, McCabe GP (2003). Introduction to the practice of statistics..

[18] Scholz J, Mannion RJ, Hord DE, Griffin RS, Rawal B (2009). A novel tool for the assessment of pain: validation in low back pain.. PLoS Med.

[19] Liu X, Eschenfelder S, Blenk KH, Janig W, Habler H (2000). Spontaneous activity of axotomized afferent neurons after L5 spinal nerve injury in rats.. Pain.

[20] Ochoa JL, Campero M, Serra J, Bostock H (2005). Hyperexcitable polymodal and insensitive nociceptors in painful human neuropathy.. Muscle Nerve.

[21] Serra J, Sola R, Quiles C, Casanova-Molla J, Pascual V (2009). C-nociceptors sensitized to cold in a patient with small-fiber neuropathy and cold allodynia.. Pain.

[22] Wasner G, Schattschneider J, Binder A, Baron R (2004). Topical menthol–a human model for cold pain by activation and sensitization of C nociceptors.. Brain.

[23] Baumgartner U, Magerl W, Klein T, Hopf HC, Treede RD (2002). Neurogenic hyperalgesia versus painful hypoalgesia: two distinct mechanisms of neuropathic pain.. Pain.

[24] Sandkuhler J (2009). Models and mechanisms of hyperalgesia and allodynia.. Physiol Rev.

[25] Baron R (2000). Peripheral neuropathic pain: from mechanisms to symptoms.. Clin J Pain.

[26] Maleki J, LeBel AA, Bennett GJ, Schwartzman RJ (2000). Patterns of spread in complex regional pain syndrome, type I (reflex sympathetic dystrophy).. Pain.

[27] Oaklander AL, Romans K, Horasek S, Stocks A, Hauer P (1998). Unilateral postherpetic neuralgia is associated with bilateral sensory neuron damage.. Ann Neurol.

[28] Baron R, Saguer M (1994). Axon-reflex reactions in affected and homologous contralateral skin after unilateral peripheral injury of thoracic segmental nerves in humans.. Neurosci Lett.

[29] Shenker NG, Haigh RC, Mapp PI, Harris N, Blake DR (2008). Contralateral hyperalgesia and allodynia following intradermal capsaicin injection in man.. Rheumatology (Oxford).

[30] Veldman PH, Goris RJ (1996). Multiple reflex sympathetic dystrophy. Which patients are at risk for developing a recurrence of reflex sympathetic dystrophy in the same or another limb.. Pain.

[31] Hubbard RD, Winkelstein BA (2008). Dorsal root compression produces myelinated axonal degeneration near the biomechanical thresholds for mechanical behavioral hypersensitivity.. Exp Neurol.

[32] Chacur M, Milligan ED, Gazda LS, Armstrong C, Wang H (2001). A new model of sciatic inflammatory neuritis (SIN): induction of unilateral and bilateral mechanical allodynia following acute unilateral peri-sciatic immune activation in rats.. Pain.

[33] Koltzenburg M, Wall PD, McMahon SB (1999). Does the right side know what the left is doing?. Trends Neurosci.

[34] Hansson E (2006). Could chronic pain and spread of pain sensation be induced and maintained by glial activation?. Acta Physiol (Oxf).

[35] Milligan ED, Twining C, Chacur M, Biedenkapp J, O’Connor K (2003). Spinal glia and proinflammatory cytokines mediate mirror-image neuropathic pain in rats.. J Neurosci.

[36] Gao YJ, Xu ZZ, Liu YC, Wen YR, Decosterd I (2010). The c-Jun N-terminal kinase 1 (JNK1) in spinal astrocytes is required for the maintenance of bilateral mechanical allodynia under a persistent inflammatory pain condition.. Pain.

[37] Watkins LR, Maier SF (2002). Beyond neurons: evidence that immune and glial cells contribute to pathological pain states.. Physiol Rev.

[38] Haanpaa M, Attal N, Backonja M, Baron R, Bennett M (2010). NeuPSIG guidelines on neuropathic pain assessment..

