# Supplementary figures and images for: Bilateral Sensory Abnormalities in Patients with Unilateral Neuropathic Pain; A Quantitative Sensory Testing (QST) Study
Source: PLoS One. 2012 May 22;7(5):e37524. doi: 10.1371/journal.pone.0037524 (PMC3358252; doi:10.1371/journal.pone.0037524)

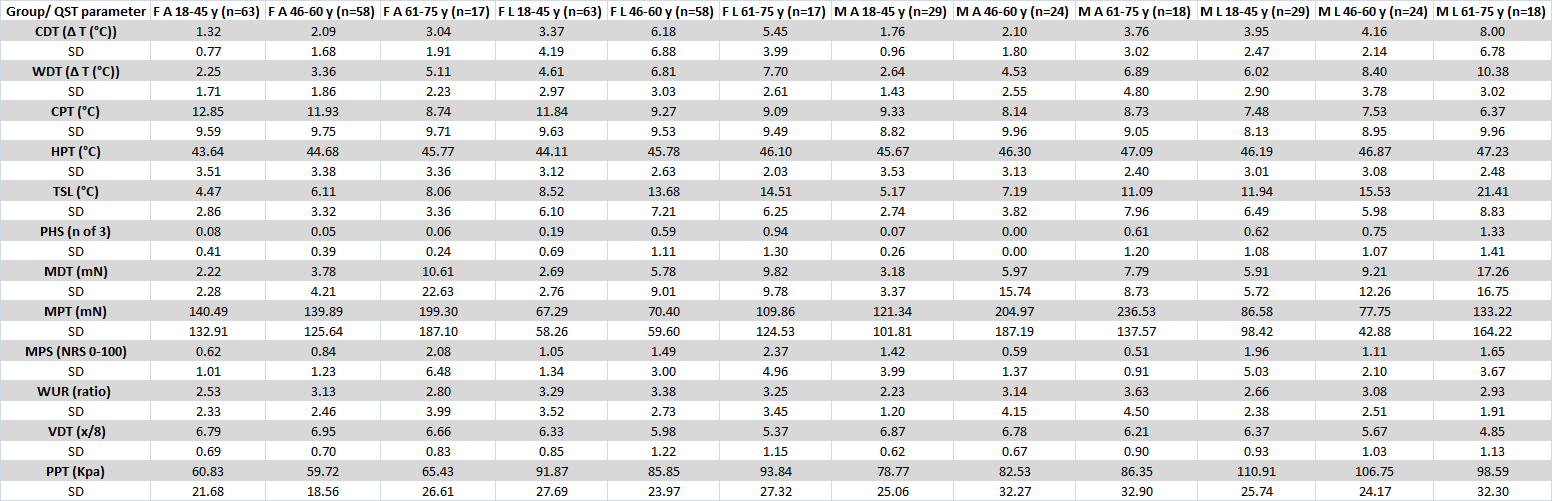

Supplement: Table S1 — Quantitative Sensory Testing normative data of healthy volunteers. Quantitative Sensory Testing (QST) normative values assessed on dorsal hand (A) and dorsal foot (L) for female (F) and male (M) according to three age groups (18–45 years (y), 46–60 years and 61–75 years of age) in healthy volunteers. QST parameters: Cold Pain Threshold (CPT), Heat Pain Threshold (HPT), Warm Detection Threshold (WDT), Wind Up Ratio (WUR) (intensity of perception of single vs. series of 1 Hz stimuli as NRS), Cold Detection Threshold (CDT), Thermal Sensory Limen (TSL), Paradoxical Heat Sensation (PHS), Mechanical Pain Threshold (MPT), Mechanical Pain Sensitivity (MPS), Mechanical Detection Threshold (MDT), Vibration Disappearance Threshold (VDT), Pressure Pain Threshold (PPT). The absence of DMA is normal and therefore described. Numeric Rating scale (NRS) indicate “0” as “no pain” and “100” as the ‘‘most intense pain imaginable’’. Unit of each QST parameter is indicated. Values indicate mean ± SD for each QST parameter. (TIF) [file pone.0037524.s001.tif]
